# Smear grading and the Mantoux skin test can be used to predict sputum smear conversion in patients suffering from tuberculosis

**DOI:** 10.3205/dgkh000297

**Published:** 2017-08-15

**Authors:** Mahmood Saffari, Hadis Alizadeh Jolandimi, Mojtaba Sehat, Nastarn Vali Nejad, Mehrdad Hedayati, Mahbobeh Zamani, Amir Ghasemi

**Affiliations:** 1 Department of Microbiology and Immunology, Faculty of Medicine, Kashan University of Medical Sciences, Kashan, Iran; 2 Trauma Research Center, Kashan University of Medical Sciences, Kashan, Iran; 3 Department of Infectious Diseases and Immunology, University of Florida, Gainesville, Florida, USA

**Keywords:** tuberculosis, sputum grading, conversion, Mantoux skin test

## Abstract

**Purpose:** Smear scores and induration sizes resulting from the PPD (tuberculin purified protein derivative) test can serve as indicators of whether a patient suffering from tuberculosis shows smear conversion or not.

**Methods:** Using microbiological methods smear and sputum tests, patients diagnosed as infected with *Mycobacterium tuberculosis* between 2002 and 2015 were included in this study. All of the assumed factors that may have a role in smear conversion were studied, in addition to the prolongation of tuberculosis.

**Results:** 398 of 512 patients fulfilled all the inclusion criteria and formed the basis of this study. 215 patients (54%) were females and 183 (46%) were males. The median age for both men and women was 36 years. We found a statistically significant difference between the size of induration resulting from the PPD skin test and the rate of non-conversion (*P*=0.002). Further univariate analysis also showed that smear grading and an induration size of ≥10 mm were independently associated with delayed smear conversion. Patients with cavitary lesions showed a higher rate of non-conversion after two months, which was not significant. We could not find any association between some of the variables, such as age, sex, weight, smoking, alcoholism, addictions, respiratory diseases, diabetes mellitus, alternative anti-TB treatment, and smear conversion.

**Conclusion:** Intensified treatment and precautions against transmission should be especially considered for TB patients with high smear grading and an induration size of more than 10 mm.

## Zusammenfassung

**Hintergrund:** Sputum-Grading und der Durchmesser der Induration nach intrakutaner Applikation von Tuberculin-purified Protein Derivate (PPD) können dafür zugrunde gelegt werden, ob ein Patient mit Tuberkulose eine Konversion im Sputum zeigt.

**Methode:** Patienten mit mikrobiologisch gesicherter Tuberkuloseinfektion im Sputum wurden zwischen 2002 und 2015 in die Studie eingeschlossen. In Ergänzung zur Erkrankungsdauer der Tuberkulose wurden alle vermuteten Faktoren, die von Einfluss auf die Sputumkonversion sein können, analysiert.

**Ergebnis:** Von 512 Patienten erfüllten 398 die Einschlusskriterien der Studie. 215 Patienten waren weiblich (54%), 183 männlich (46%). Das durchschnittliche Alter betrug 36 Jahre. Wir fanden einen statistisch signifikanten Unterschied zwischen dem Durchmesser der Induration des PPD-Hauttests und der Rate der Nicht-Konversion (*P*=0.002). Im Ergebnis der univariaten Analyse waren Sputum-Grading, Durchmesser der Induration von ≥10 mm und größere Schwellung unabhängig assoziiert mit verzögerter Sputumkonversion. Patienten mit kavitären Läsionen zeigten eine höhere, aber nicht signifikante non-Konversionsrate nach 2 Monaten. Es war kein Zusammenhang zu Alter, Geschlecht, Körpermasse, Rauchen, Alkoholismus, Suchtverhalten, respiratorischen Erkrankungen, Diabetes mellitus, alternativer antituberkulöser Behandlung und Sputumkonversion nachweisbar.

**Schlussfolgerung:** Eine intensivierte Therapie und Vorsichtsmaßnahmen zur Verhinderung einer Übertragung sollten besonders für Tuberkulosepatienten mit hohem Sputum Grading und Induration >10 mm berücksichtigt werden.

## Introduction

Tuberculosis (TB) is a bacterial infection caused by *Mycobacterium tuberculosis* [1]. In spite of global endeavors to eradicate TB, it is still a public health problem world-wide, especially in developing countries [2]. Over the past several decades, there have been continuous efforts to achieve an efficacious treatment of TB [3]. The goal of tuberculosis treatment is to diminish the spread of the infection, and the most effective method for avoiding transmission is the identification and treatment of patients suffering from infectious pulmonary tuberculosis [4]. Patients with a failed treatment outcome would be a public health risk, as these patients may have developed drug resistance and remain infectious for a long time [5]. TB treatment results are affected by drug-resistant bacteria, patient characteristics, patient actions, and quality of health care. Hence, the surveillance of treatment is paramount in the Directly Observed Treatment Short-course (DOTS) strategy [6]. The results of the follow-up, using a microbiological examination at the end of the two-month intensive phase of treatment, provide an indication of whether the therapeutic regimen can be shifted to the perpetuation phase, or whether the patient should be given an extra month of intensive phase treatment. However, it has been reported that the results of sputum smear-positive pulmonary tuberculosis obtained from patients, at the end of two months of anti-tuberculosis treatment, can predict unexpected outcomes in terms of increased failure of treatment or relapse rates of tuberculosis [7], [8], [9]. Awareness of the factors related to persistent sputum positivity at the end of two months of anti-tuberculosis treatment might, therefore, help clinicians to better manage their patients and improve outcomes. Thus, we decided to examine all of the assumed risk factors that can impact the smear conversion in sputum following two months of therapy, in addition to the consequent prolongation of TB during a period of 12 years in Kashan County in central Iran. Although the interferon γ release assay (IGRA) was approved by the Food and Drug Administration (FDA) as means of detecting *M. tuberculosis* infection in 2001 [10] and has become common in clinical use in some contexts instead of the Mantoux skin test [11], the WHO has strongly recommended that IGRAs should not be used in low- and middle-income countries for the detection of pulmonary or extra-pulmonary TB [12]. Thus, in our study, the Mantoux test was only considered for diagnosis of TB infection.

## Materials and methods

### Study population

In a retrospective cohort study, all first-time patients who referred to the Kashan Health Centers from January 2002 to December 2014 were included for analysis. All patients were enrolled, treated, and managed according to the procedures of the National Tuberculosis Control Plan. All of the patients received standardized treatment directly after enrollment. 

The medical records of enrolled patients were reviewed to obtain their microbiological examinations, co-comorbidities, information on smoking, HIV status, clinical presentation, the results of the PPD skin test, an anti-TB treatment regimen, and demographic as well as therapeutic data. Ethical permission was obtained from the Kashan University of Medical Sciences Ethics Committee. The standardized treatment regimen consists of two months of thrice-weekly isoniazid, pyrazinamide, rifampicin, ethambutol, and streptomycin administration, followed by four months of thrice-weekly administration of isoniazid, rifampicin, and ethambutol.

### Microbiological and molecular tests

In order to determine whether bacteria were present in the sputum, the relevant standard procedures were performed as follows: NaOH was used to decontaminate early-morning sputum samples. Smears were prepared using the Ziehl-Neelsen technique, then examined under oil immersion using a conventional microscope for 3 consecutive days for each suspected patient. Findings were scored as follows: 1–9AFB/100 fields (1+); 1–9AFB/10fields (2+); and1–9AFB/field (3+) (Thoracic Society guidelines [13]). To culture mycobacteria, solid culture media were used, such as 7H10 or 7H11 and Lowenstein–Jensen media [14]. In addition, biochemical and molecular tests were used to further confirm *M. tuberculosis* [15]. DNA typing, including RFLP, was used for final identification [16], [17], [18]. Susceptibility of isolates to isoniazid, rifampin, pyrazinamide, ethambutol, and streptomycin were determined as recommended by the CLSI (Clinical and Laboratory Standards Institute; formerly National Committee for Clinical Laboratory Standards – NCCLS) using the standard proportion method on a 7H10 agar medium. All sputum smears and cultures were tested two and five months after the beginning of treatment. Smear conversion was described as 2 repeated negative samples. Delayed smear and culture conversion were described as persistent positivity after 2 months of treatment. The sputum with delayed smear conversion was cultured to obtain further confirmation. On a daily basis, all patients received intensive-phase antimicrobial treatment, including rifampicin, pyrazinamide, isoniazid, and ethambutol over the course of the first 2 months. Rifampicin, isoniazid, and ethambutol were administered during a 4-month continuation phase [19]. However, the intensive phase was prolonged by an additional month if the smear and culture were positive. 

### Skin test

The Mantoux test [20] was performed by an experienced physician injecting 0.1 ml of tuberculin-purified protein derivative (PPD) into the skin of the inner surface of the forearm. After measuring two vertical diameters of the skin induration, the average was obtained for interpretation. Indurations were scored as no response or energy (0 mm induration diameter), negativity (0 to 4 mm induration diameter), reactivity (5 to 9 mm induration diameter), and positivity (≥10 mm induration diameter) [21].

### Statistical analysis

SPSS version 16.0 software was used for statistical analysis. One-way ANOVA was employed for between-group comparisons. *P*-values <0.05 were considered significant. Fisher’s exact test or the chi-squared test was used to compare categorical variables whenever appropriate. Delayed sputum conversion was a dichotomous, dependent variable. Statistically significant variables in univariate analysis were used in a logistic regression model to examine which factors were independently and significantly associated with the dependent variable. The odds ratios (OR) and 95% confidence intervals (CI) were calculated.

## Results

From January 2002 to December 2014, 512 new enrolled patients were identified as having pulmonary TB. Of these patients, 398 fulfilled all the inclusion criteria and formed the basis of this study. The other 114 patients were classified as outpatients due to death during hospitalization, moving back to their home country (Afghanistan) during the first part of treatment, and failure to follow-up after discharge from the hospital. 215 patients (54%) were female and 183 (46%) were male. Patient ages ranged between 18 and 68 years (Table 1 (Tab. 1)). The median age for both men and women was 36 years. 274 (69.8%) patients had respiratory distress at treatment initiation and 167 (43.2%) patients had cavities in their lungs. Comorbidities were present in 167 (42.0%) patients. Only two (0.5%) patients had HIV co-infection. The rate of sputum conversion at the second month of treatment was 84% (338 patients) (Table 1 (Tab. 1)). Among patients who had sputum smear conversion, the average time to sputum conversion was 62 days. There were no statistically significant differences in other analyzed variables, such as age, sex, weight, smoking, alcoholism, addictions, respiratory diseases, diabetes mellitus, alternative anti-TB treatment, and related toxicity. Influences such as the initial bacterial load, chemotherapy regimen, gender, and different age groups on sputum conversion were also analyzed. Although no significant difference was noticed in either gender or in the different age groups, patients older than 60 showed a good sputum conversion compared to those who were 40 to 60 years old, although this difference was not statistically significant. There was no difference in the rate of sputum conversion of patients infected with drug-susceptible strains, regardless of receiving ethambutol in addition to rifampicin, isoniazid, and pyrazinamide. 

Although the association between cavitation and sputum conversion was not statistically significant, cavities were most frequently seen in the smear-positive group. 

Surprisingly, the data obtained from the PPD experiment showed that there is a statistically significant difference between the size of induration resulting from the PPD skin test and the rate of non-conversion (*P*=0.002). Twenty percent of patients with delayed sputum conversion showed skin test induration sizes of 5 to 9 mm, while 72 percent of patients had more than 10 mm of induration swelling. 

Multivariate logistic regression analysis showed that smear scores and induration size of 10 mm or more were independently associated with delayed smear conversion (smear score 3+: OR 9.8, 95% CI 5.6–12.1; induration size: 3.16 mm (2.02–6.73).

## Discussion

Although ongoing efforts are being made to find tools to screen the treatment of tuberculosis patients and predict its consequences, studies have shown that the delayed conversion of positive smears at the end of a two-month treatment period is one of the most powerful indicators of possible treatment failure [22], [23], [24], [25]. This study is a retrospective assessment of a number of registered and treated TB cases at the Kashan Health Centers. At first, a culture-based examination was not used to confirm TB, and other tests were considered, including clinical signs, chest radiographs, and PPD skin tests. For suspected patients, sputum smear and culture examination of TB were used. Following this, TB patients were confirmed through molecular methods. In total, 512 patients were found to be infected with TB and were considered for this study. However, 114 patients were omitted from the study, as complete information was not accessible due to either death, moving back to their home country, or poorly recorded data. Nineteen percent of patients showed non-conversion of sputum, first demonstrated through a smear examination and further confirmed by culturing. We then evaluated the factors that might influence the rate of conversion. The present study found no association between the following factors: gender, age, weight, smoking, alcoholism, addictions, respiratory diseases, diabetes mellitus, alternative anti-TB treatment, and toxicity related to the outcome of the disease. Such findings were similar to the results of other studies [26]. Although some reports exist on an association between comorbidities, such as diabetes and conversion rate [27], we did not find such an association. This is probably due to the small patient population of this study with morbidities such as diabetes. However, the present study is comparable with other studies, having shown there is no association between diabetes and sputum smear conversion [28], [29]. Conversely, smoking was shown to be associated with sputum smear conversion after 2 months of anti-tuberculosis treatment [30]. Patients with cavitary lesions have demonstrated a higher rate of non-conversion [31], [32], although this association was not statistically significant in the present study. A possible bias due to chest radiograph interpretation may be the reason for such a finding. Scoring of sputum smears is a widely applied tool to detect tuberculosis in patients, and can also serve to indicate the cure rate and quality of sputum smear examination [33]. This test enables detection of a major proportion of undiagnosed cases, especially among females. Around 10% of diagnostic smears and 50% of follow-up smears were found to be light smears [34].

This study found that the bacterial load was significantly associated with the non-conversion rate. In this regard, several studies have reported that smear-positive patients with a high bacterial load showed statistically significantly poor sputum conversion rates at two and three months, and higher failure rates as compared to patients with a lower bacterial load [22], [24], [25], [35], [36], [37], [38]. The current study was limited by the fact that microscopy was performed at different laboratories using local criteria for AFB scoring, and this could have caused some miscategorization. However, scoring criteria were well verified and it was thus unlikely that such miscategorization affected the study results. 

A tuberculin skin test or Mantoux tuberculin test is performed to determine whether an individual has ever been exposed to TB. The test is done by injecting a small amount of PPD antigen under the top layer of skin. The tuberculin most broadly used is purified protein derivative (PPD) derived from cultures of *M. tuberculosis*. The “old tuberculin” is no longer used for this purpose; in its place, a much more standardized product termed PPD-S (purified protein derivative, prepared according to the method described by Siebert) from *M. tuberculosis* is used [39]. If there is any infection with *M. tuberculosis*, the skin will react to PPD by forming a firm red knot at the site within 2 days [11]. The PPD reaction utilizes the delayed-type hypersensitivity reaction of a patient before being infected with mycobacteria [40]. A positive reaction is histologically accompanied by the existence of mononuclear cells at the position of injection. In spite of its poor specificity, the PPD test is still thought to be useful for identifying TB infection in non-HIV-infected individuals [20]. Based on our findings, patients with induration swelling of more than 10 mm developed a higher delayed sputum conversion compared to patients with 5 to 9 mm of skin test induration sizes. PPD reactivity is based on the secretion of cytokines, which cause an inflammatory reaction due to T cells being exposed to mycobacterial antigens *in vivo*, as well as the characteristic induration caused by the infiltration of PPD-specific lymphocytes, resulting in a local inflammatory response at the site of injection [41]. Therefore, it is conceivable that if a greater bacterial load existed, a larger induration size would be expected, which was also shown in the smear scores in this study.

## Conclusion

Although it has been reported that many factors lead to smear conversion, the present analysis showed that smear scoring and induration sizes of more than 10 mm from the PPD test were associated with delayed smear conversion but other factors, including sex, age and comorbidity are not the risk factors in delay in smear conversion. We suggest that intensified treatment and precautions against transmission should be considered for TB patients who possess these risk factors, as this would subsequently allow the optimization of national TB control measures. Moreover, additional prospective studies on sputum conversion and culture conversion are needed to consolidate the current findings.

## Notes

### Competing interests

The authors declare that they have no competing interests.

### Financial disclosure

This work was supported by grants from Kashan University of Medical Sciences (grant no. 93194).

## Figures and Tables

**Table 1:**
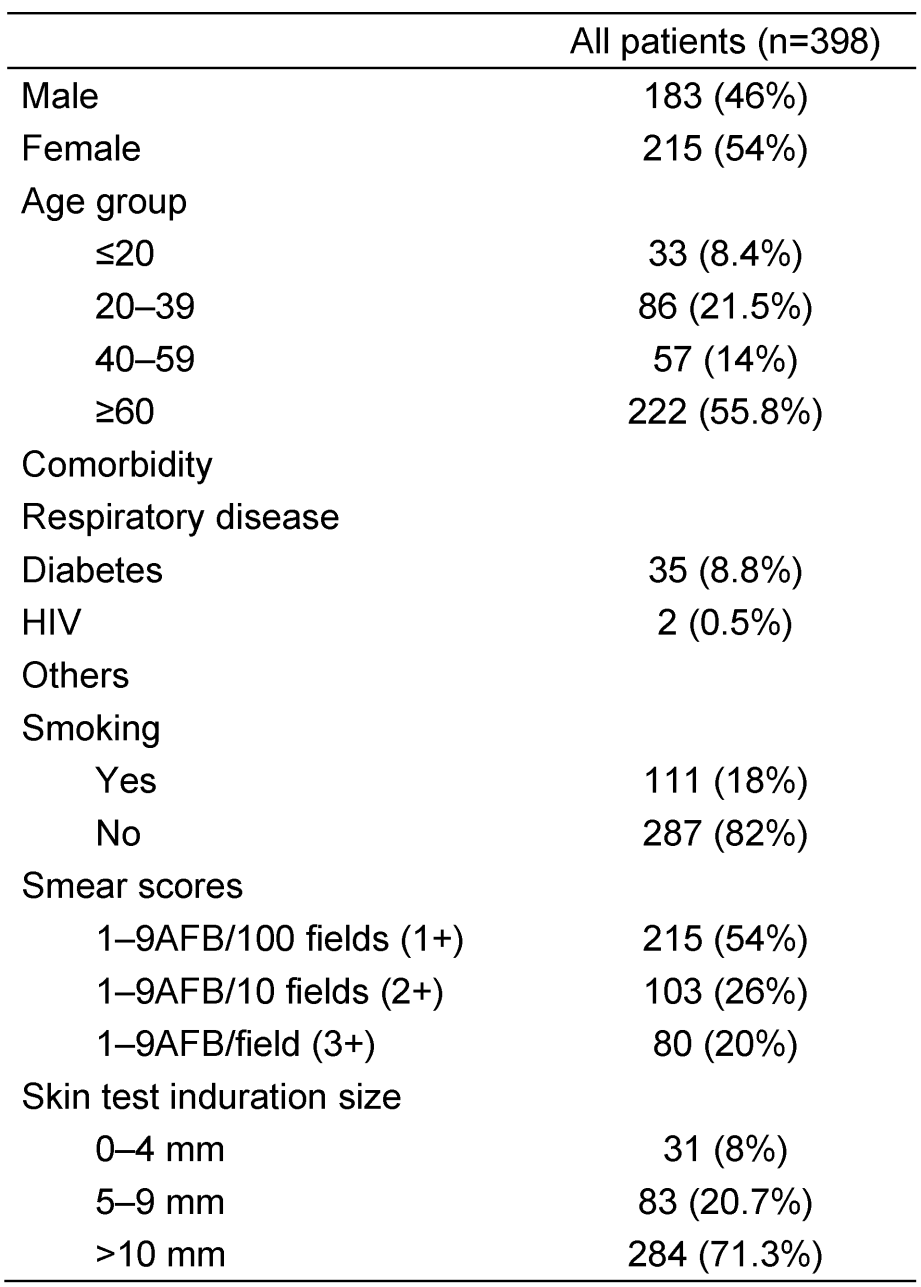
Patient characteristics
